# The preliminary study of prebiotic potential of Polish wild mushroom polysaccharides: the stimulation effect on *Lactobacillus* strains growth

**DOI:** 10.1007/s00394-017-1436-9

**Published:** 2017-03-28

**Authors:** Renata Nowak, Natalia Nowacka-Jechalke, Marek Juda, Anna Malm

**Affiliations:** 1 Chair and Department of Pharmaceutical Botany, Medical University of Lublin, 1 Chodźki Street, 20-093 Lublin, Poland; 2 Department of Pharmaceutical Microbiology, Medical University of Lublin, 1 Chodźki Street, 20-093 Lublin, Poland

**Keywords:** Polysaccharides, Prebiotics, *Lactobacillus*, Nutraceuticals, Functional foods

## Abstract

**Purpose:**

According to the vast body of evidence demonstrating that the intestinal microbiota is undoubtedly linked with overall health, including cancer risk, searching for functional foods and novel prebiotic influencing on beneficial bacteria is necessary. The present study aimed to investigate the potential of polysaccharides from 53 wild-growing mushrooms to stimulate the growth of *Lactobacillus acidophilus* and *Lactobacillus rhamnosus* and to determine the digestibility of polysaccharide fractions.

**Methods:**

Mushroom polysaccharides were precipitated with ethanol from aqueous extracts. Determination of growth promoting activity of polysaccharides was performed in U-shaped 96-plates in an ELISA reader in relation to the reference strain of *L. acidophilus* and two clinical strains of *L. rhamnosus*. The digestibility of mushroom polysaccharides was investigated *in vitro* by exposing them to artificial human gastric juice.

**Results:**

Obtained results revealed that fungal polysaccharides stimulate the growth of *Lactobacillus* strains stronger than commercially available prebiotics like inulin or fructooligosaccharides. Moreover, selected polysaccharides were subjected to artificial human gastric juice and remain undigested in more than 90%.

**Conclusion:**

Obtained results indicate that mushroom polysaccharides are able to pass through the stomach unchanged, reaching the colon and stimulating the growth of beneficial bacteria. Majority of 53 polysaccharide fractions were analysed for the first time in our study. Overall, our findings suggest that polysaccharide fractions from edible mushrooms might be useful in producing functional foods and nutraceuticals.

**Electronic supplementary material:**

The online version of this article (doi:10.1007/s00394-017-1436-9) contains supplementary material, which is available to authorized users.

## Introduction

Mushrooms constitute a huge and underappreciated source of agents with extensive health benefits. There are almost 150,000 species all over the world, only approximately 5% of which have been investigated for medicinal or nutritional properties [1, 2]. Eastern Asian countries possess long traditions of using mushrooms for both edible and medicinal purposes. In China, fungi have been used as a dietary supplement or medicinal food for 2000 years [3]. There is a vast body of evidence indicating that mushrooms demonstrate immunomodulating, antiviral, antidiabetic, antitumor, antioxidant, antibacterial and hypocholesterolemic effects [4–7]. These activities result from the presence of biologically active constituents, including polysaccharides, which have recently generated growing interest.

Mushroom polysaccharides are structural components of cell walls which can be divided into two major types: rigid fibrillars of chitin and the more abundant glucans. Glucans include β-glucans with variable proportion of β-1,3 and β-1,6 linkages as well as α-1,3-glucans [8]. β-Glucans, also present in cereal crops like barley and oats, are considered a valuable functional ingredient of many food products due to their nutritional and health benefits as well as their use as thickening or stabilizing agents and in gelation. Several applications of β-glucans in food products including bread, dairy products, breakfast cereals or beverages are known [9]. Purified β-glucans can be incorporated into wheat flour used in the production of pasta products, bread and other bakery products, enriching them in dietary fibre. Moreover, they can be used as fat replacers in low-fat yoghurts, ice creams, cheeses and butter-like products by enhancing the rheological, textural and sensory properties of food products [10–12]. Therefore, the incorporation of polysaccharides, especially glucans of natural origin, into different food systems is valuable and of potential interest worldwide.

During recent decades, there has been an increased awareness of food effects on health. Several epidemiological studies have demonstrated that functional foods reduce the risk of cancer, improve heart health, enhance the immune system and improve gastrointestinal (GI) health [11]. The concept of functional food includes prebiotics, defined as non-digestible food ingredients stimulating the growth of beneficial bacteria in the GI tract. The main criterion for potential prebiotic agents is that they must pass to the large intestine without being digested or absorbed in the upper GI tract, so that they become accessible for probiotic bacteria [13, 14]. Prebiotics have been found to reduce the incidence and duration of intestinal infections, down-regulate allergic response, improve digestion and improve the elimination of faeces. Prebiotics’ beneficial effect is associated with changes in the composition of GI microbiota. Positive prebiotic activity of some substances results in significant increases in the numbers of bifidobacteria and lactobacilli [3, 15–17]. These two genera exert wide-range activity; bifidobacteria have been shown to stimulate the immune system, restore the normal flora after antibiotic therapy, inhibit pathogen growth and produce vitamin B, while lactobacilli help to digest lactose in lactose-intolerant individuals, as well as reduce constipation, diarrhoea or irritable bowel syndrome [3].

Non-digestible carbohydrates, including di-, oligo- and polysaccharides, galactooligosaccharides and fructooligosaccharides as well as inulin have been studied most extensively and are considered the best prebiotics. These compounds can be obtained from different natural sources including chicory, asparagus, artichoke, onion, garlic and other plants [11]. Given the growing demand for prebiotics, new and relatively cheap sources compared to commercially available prebiotics must be found.

As mentioned above, mushrooms are also a very rich source of polysaccharides; therefore, the aim of our study was to investigate if fungal polysaccharides extracted from various wild-growing Polish species might act as growth substrates for the reference *Lactobacillus acidophilus* strain and two clinical strains of *Lactobacillus rhamnosus*. Moreover, the digestibility of the selected mushroom polysaccharides was determined by calculating their degree of hydrolysis upon exposure to artificial human gastric juice. To the best of our knowledge, this report describes the first such wide-range screening study on the stimulation of probiotic bacteria growth by polysaccharides from Polish wild mushrooms and it constitutes preliminary study investigating if these compounds might comply with one of the prebiotic criteria.

## Materials and methods

### Materials

The wild-growing fruiting bodies of *Amanita citrina* (Schaeff.) Pers., *Amanita muscaria* (L.: Fr.) Hook., *Amanita pantherina* (DC.: Fr.) Krombh., *Armillaria mellea* (Vahl: Fr.) P. Kumm. ss. lato., *Bjerkandera adusta* (Willd.: Fr.) P. Karst., *Clavicorona pyxidata* (Pers.: Fr.) Doty, *Clavulina cinerea* (Bull.: Fr.) J. Schröt, *Clitocybe gibba* (Pers.: Fr.) P. Kumm., *Clitocybe nebularis* (Batsch: Fr.) P. Kumm., *Coprinus micaceus* (Bull.: Fr.) Fr., *Cortinarius cinnamomeus* (L.: Fr.) Fr., *Cortinarius sanguineus* (Wulf.: Fr.) Fr., *Daedaleopsis confragosa* (Bolt.: Fr.) J. Schröt., *Fomes fomentarius* (L.: Fr.) Kickx, *Fomitopsis pinicola* (Swartz.: Fr.) P. Karst., *Ganoderma applanatum* (Pers.) Pat., *Gymnopilus penetrans* (Fr.: Fr.) Murrill, *Gymnopus dryophilus* (Bull. Fr.) Murrill, *Heterobasidion annosum* (Fr.) Bref. ss. Lato, *Hygrophoropsis aurantiaca* (Wulf.: Fr.) J. Schröt., *Hyphodontia paradoxa* (Schrad.: Fr.) E. Langer & Vesterholt ss. Lato, *Laccaria amnethystina* (Bull.) Murrill, *Laccaria laccata* (Scop.: Fr.) Berk. & Broome, *Lactarius aurantiacus* (Pers.: Fr.) Gray, *Lactarius helvus* (Fr.) Fr., *Lactarius resimus* (Fr.: Fr.) Fr., *Lactarius rufus* (Scop.: Fr.) Fr., *Lactarius vellereus* (Fr.) Fr., *Laetiporus sulphureus* (Bull.: Fr.) Murrill, *Leccinum scabrum* (Bull.: Fr.) Gray, *Lepista flaccida* (Sowerby: Fr.) Pat., *Lycoperdon perlatum* Pers.: Pers., *Macrolepiota procera* (Scop.: Fr.) Singer, *Marasmius oreades* (Bolt.: Fr.) Fr., *Morchella conica* Pers., *Panellus stypticus* (‘stipticus’) (Bull.: Fr.) P. Karst., *Paxillus involutus* (Batsch: Fr.) Fr. Ss. lato, *Pholiota mutabilis* (Scop.: Fr) P. Kumm., *Piptoporus betulinus* (Bull.: Fr.) P. Karst., *Pseudoclitocybe cyanthiformis* (Bull.: Fr.) Singer, *Psilocybe capnoides* (Fr.: Fr.) Noordel., *Psilocybe fascicularis* (Huds.: Fr.) Noordel., *Psilocybe lateritia* (Schaeff.: Fr.) Noordel., *Rhodocollybia maculata* (Alb. & Schwein.: Fr.) Singer, *Russula fragilis* (Pers.: Fr.) Fr. var fragilis, *Scleroderma citrinum* Pers., *Sparassis crispa* (Wulf.: Fr.), *Suillus bovinus* (L.: Fr.) Roussel, *Suillus variegatus* (Schwein.: Fr.) O. Kuntze, *Trametes hirsuta* (Wulf.: Fr.) Pilát, *Trametes versicolor* (L.: Fr.) Pilát, *Tubaria furfuracea* (Pers.: Fr.) Gillet and *Xerocomus badius* (Fr.: Fr.) Kühner ex Gilbert were collected in the forests near Włodawa (Lublin Voivodeship, Poland) in September 2014. Mushroom specimens were authenticated by Dr. Zofia Flisińska from the Department of Botany and Mycology, Maria Curie-Skłodowska University, Lublin, Poland. Voucher specimens were deposited at the Department of Pharmaceutical Botany, Medical University of Lublin, Poland. The mushrooms were immediately lyophilized in a Free Zone 1 apparatus (Labconco, Kansas City, KS, USA), pulverized and kept in a freezer (−30 °C) until further analysis.

### Chemicals

Inulin and fructooligosaccharides (FOS) from chicory, glucose and phenol were purchased from Sigma-Aldrich Fine Chemicals (St. Louis, MO, USA). Tryptone, yeast extract and Tween 80 were purchased from POCH (Gliwice, Poland). All other chemicals and solvents were of analytical grade and were purchased from POCH (Gliwice, Poland).

### Polysaccharide extract preparation

The freeze-dried and milled fruit bodies of mushrooms (5 g) were extracted twice with 99.8% ethanol (50 mL each time) by shaking for 24 h at room temperature to remove low molecular weight substances. The supernatants were removed and the residues were dried and macerated twice with hot water (50 mL) by shaking for 1 h. Then the supernatants were removed and the residues were extracted two times for 30 min with distilled water (100 mL each time) under sonication at 80 °C. The combined aqueous extracts were concentrated under vacuum to 10 mL. The concentrated extracts were further purified by deproteinization using the Savage reagent (chloroform/isoamyl alcohol, 4:1, v/v). Subsequently, the polysaccharides were precipitated with 4 volumes of cold 99.8% ethanol and kept overnight in the refrigerator at 4 °C. The resulting precipitates were collected by centrifugation (9000 rpm, 15 min) and lyophilized in a Free Zone 1 apparatus (Labconco, Kansas City, KS, USA). The percentage polysaccharide yields (%) were calculated as follows:

$${\rm{Yield }}\left( {\% ,{\rm{ w}}/{\rm{w}}} \right){\rm{ }} = {\rm{ Weight\,of\,extracted\,polysaccharides}}/{\rm{Weight\,of\,dried\,material }} \times {\rm{ 1}}00$$

### Bacterial strains and growth conditions

*Lactobacillus acidophilus* ATCC 4356 (LGC Standards) and two strains of *Lactobacillus rhamnosus* isolated from the GI tract of healthy patients were used. The strains were cultivated on Rogosa agar (Oxoid) with glucose under anaerobic conditions. The Rogosa medium was prepared at pH = 5.4 ± 0.2 with the following ingredients (in g/L): tryptone—10; yeast extract—5; Tween 80—1; potassium dihydrogen phosphate—6; ammonium citrate—2; sodium acetate anhydrous—17; magnesium sulphate—0.57; manganese sulphate—0.12; ferrous sulphate—0.034. For Rogosa medium with glucose, 20 g of this carbohydrate was added. The Rogosa agar also contained 20 g agar. GENbag Anaer atmospheric generators (bioMerieux) were used for incubation under anaerobic conditions.

### Determination of probiotic bacteria growth stimulation

The modified method by Su, Henrikson and Mitchell [18] was used to determine the growth of lactobacilli in the presence of mushroom polysaccharides. The test was performed in U-shaped 96-plates in an ELISA reader (BioTek). Rogosa broth media (200 µL) without glucose was supplemented with 1.5% polysaccharide inoculated with 20 µL of *L. acidophilus, L. rhamnosus* 1 or *L. rhamnosus* 2 (according to the McFarland standards, 0.5 contains 150 × 10^6^ CFU/ml) and the plates were incubated for 72 h. The absorbance was measured after 0, 24, 48 and 72 h of incubation using an ELISA reader at a wavelength of 600 nm. Two controls were used: a negative control containing Rogosa broth without glucose supplemented by polysaccharide, and a positive control containing Rogosa broth with glucose and the *Lactobacillus* strain. The absorbance readings for each sample were compared to that of the negative control. The results were presented as the percentage of lactobacilli growth in the presence of each polysaccharide in comparison with the growth in the glucose-containing medium (taken as 100%).

### Resistance toward acid digestibility

The digestibility of the mushroom polysaccharides was tested by calculating their degree of hydrolysis when subjected to artificial human gastric juice imitated by the hydrochloric acid buffer containing (in g/L): sodium chloride—8; potassium chloride—0.2; di-sodium hydrogen phosphate dihydrate – 8.25; sodium phosphate anhydrate—14.35; calcium chloride dihydrate—0.1; magnesium chloride hexahydrate—0.18, adjusted to pH 1 and pH 5 with the addition of 5 M HCl [19]. The buffer was added to the sample solution (1% w/v) in the ratio 1:1 and incubated at 37 ± 1 °C for 2 h. FOS and inulin were used as controls. Samples were taken at 0 and 2 h and tested for reducing sugar content using a modified dinitrosalicylic acid (DNS) assay [20] and for total sugar content using the phenol–sulfuric acid method [21]. The percent hydrolysis of the sample was calculated based on the reducing sugar released and the total sugar content of the sample as below:

% hydrolysis = reducing sugar released/total sugar content − initial reducing sugar content × 100; where reducing sugar released is the difference between its final and initial content [22].

### Statistical analyses

Statistical analyses of the stimulation of the growth of lactobacilli were performed after 48 h of incubation. The results of absorbance between two groups were analysed: the Rogosa broth without glucose, containing the reference strain and supplemented with polysaccharide, was compared to the negative control. Student’s *t* test was used in statistical analyses of the results. The differences were statistically significant when *p* ≤ 0.05. The statistical analyses were performed by STATISTICA software version 10.

## Results

### Polysaccharide extraction

In our study, an optimized multi-step extraction was used. First, 99.8% ethanol was used to remove small molecules from the fruiting bodies of the mushrooms, followed by the maceration with hot water and then main extraction with hot water by sonication. The application of the Savage reagent led to removal of free proteins in order to ensure the purity of the extracted polysaccharides.

**Fig. 1:**
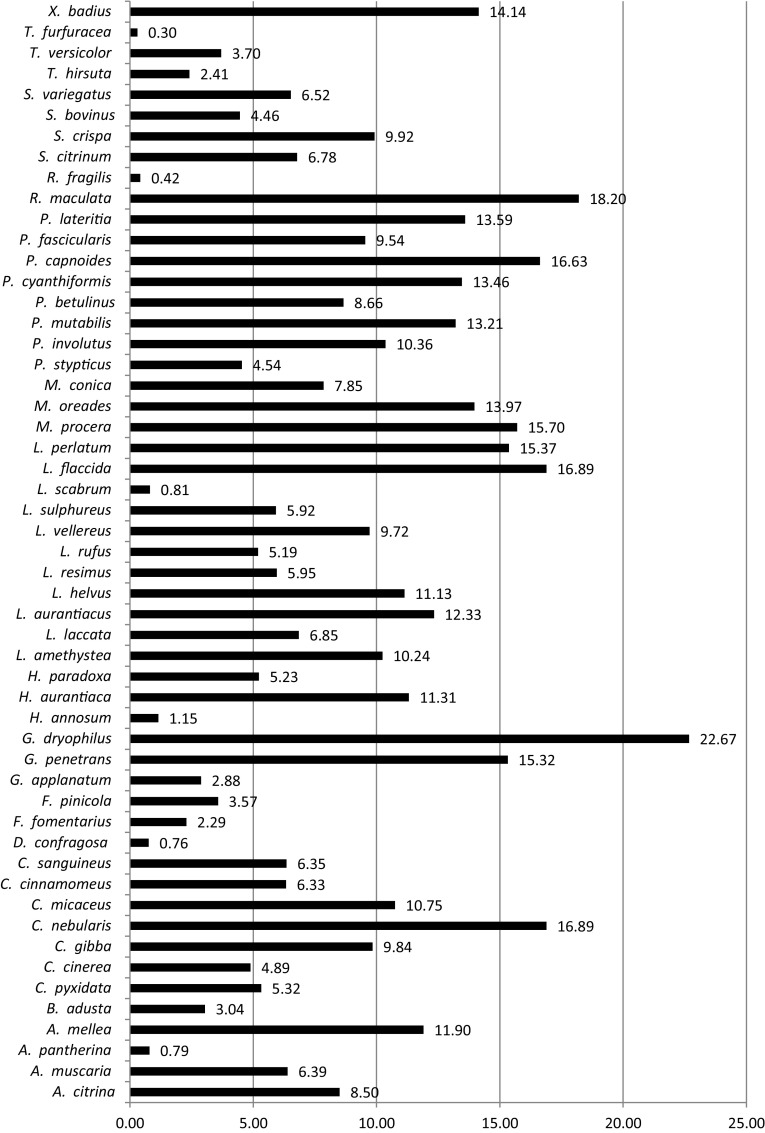
Yields of mushroom polysaccharide extractions [%]

The percentage yields of polysaccharides extractions from the investigated species are shown in Fig. 1. These yields range widely from 0.3% for *T. furfuracea* to 22.67% for *G. dryophilus*. Relatively high values, above 15%, were obtained for *R. maculata, L. flaccida, P. capnoides, M. procera* and *L. perlatum*.

### Probiotic bacteria growth stimulation

**Fig. 2:**
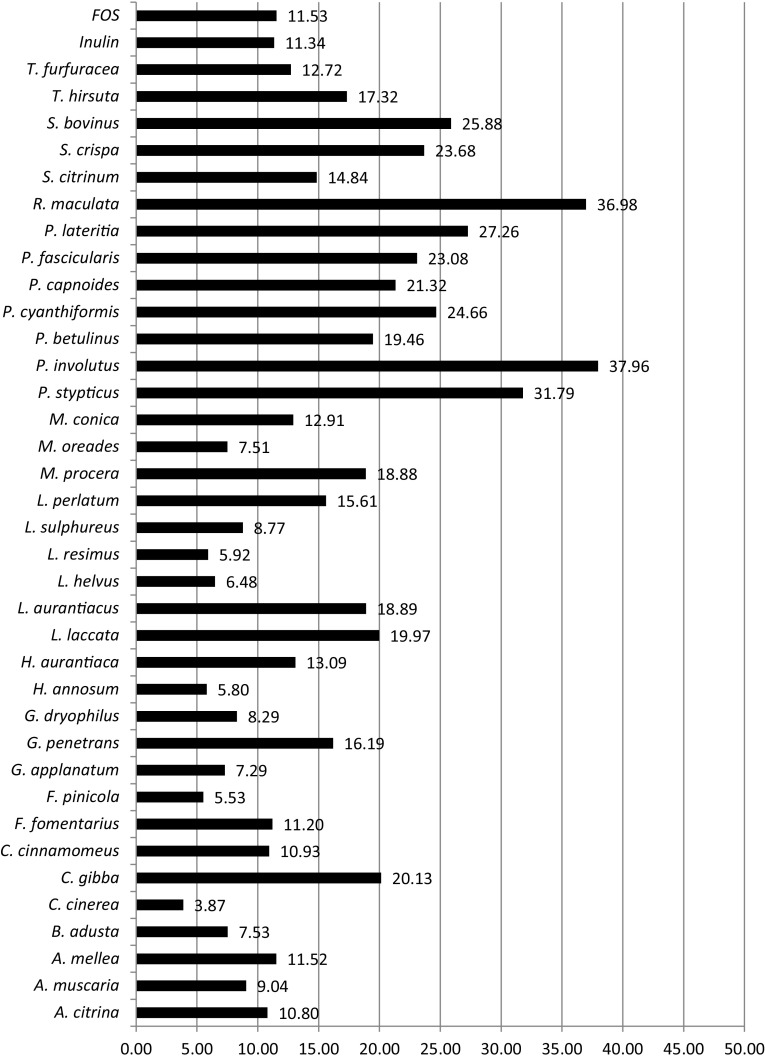
Growth stimulation activity of mushroom polysaccharides with respect to *L. acidophilus* [%]. The values were calculated on the basis of glucose activity (taken as 100%) for the species that demonstrated statistical significance (*p* ≤ 0.05) in Student’s *t* test

The ability of mushroom polysaccharides to promote the growth of lactobacilli was determined *in vitro* by measuring absorbance at 0, 24, 48 and 72 h of incubation. After 48 h, the maximum growth of *L. acidophilus* and *L. rhamnosus* was observed, and the values obtained were accepted for further statistical analyses. The growth of the reference strain *L. acidophilus* in the presence of selected polysaccharides over the incubation time is presented in Figure S1 (Online Resource 1). Among the 53 mushroom polysaccharides examined, 11 did not stimulate the growth of *L. acidophilus* and were not subjected to statistical analysis. The absorbance values for mushroom polysaccharides, inulin, FOS and controls after 48 h, as well as their respective *p-*values, are given in Table S1 (Online Resource 1). Species with *p* ≤ 0.05 were considered statistically significant; the percentage growth intensity for these polysaccharides was calculated based on the glucose activity, which was taken as 100%. Figure 2 presents the influence of the polysaccharides on the growth of reference strain *L. acidophilus*. The majority of the polysaccharides studied revealed significant activity; the values obtained for these 36 species varied from 3.87% for *C. cinerea* to 37.96% for *P. involutus*. The polysaccharides from different mushroom species could be divided into three groups according to the percentage of activity. The first group, exhibiting activity below 15%, contains 19 species. The second group, with activities ranging from 15 to 30%, includes the following species: *C. gibba, G. penetrans, L. laccata, L. aurantiacus, L. perlatum, M. procera, P. betulinus, P. cyanthiformis, P. capnoides, P. fascicularis, P. lateritia, S. crispa, S. bovinus* and *T. hirsute*. The third group, with activities above 30%, comprises *P. stypticus, P. involutus* and *R. maculata*. Species such as *G. penetrans, L. aurantiacus, L. perlatum, M. procera, P. cyanthiformis, P. capnoides* and *P. lateritia*, constitute rich sources of polysaccharides, according to the yields of the mushroom polysaccharide extractions presented in Fig. 1. Edible mushrooms are particularly interesting due to their potential in the development of nutraceutical foods. *M. procera, S. crispa* and *S. bovinus* are very common and easily accessible in nature. Moreover, they are appreciated for their taste and nutritional value, which makes them particularly interesting considering their growth substrate content. In comparison with the polysaccharides, the commercially available prebiotics FOS and inulin exhibited significantly lower level of enhancing lactobacilli growth: 11.53% and 11.34%, respectively.

**Fig. 3:**
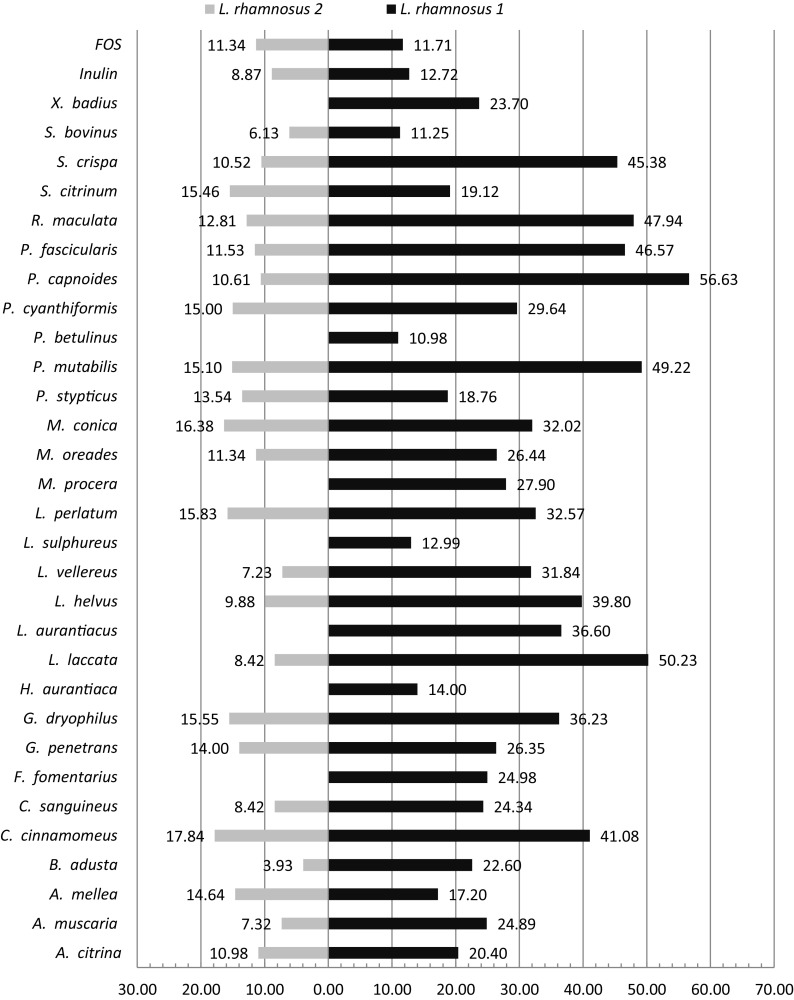
Growth promoting activity of mushroom polysaccharides with respect to *L. rhamnosus* 1 and 2 [%]. The values calculated on the basis of glucose activity (taken as 100%) for the species demonstrated statistical significance (*p* ≤ 0.05) in Student’s *t* test; those without significant prebiotic activity were not evaluated

The next step of our study was the assessment of the influence of mushroom polysaccharides in relation to growth of two strains of *L. rhamnosus* isolated from the gastrointestinal tracts of healthy people. The results are presented in Fig. 3. Polysaccharides were selected on the basis of previous determinations with the reference strain *L. acidophilus*. Among the 33 samples examined, only two did not stimulate the growth of *L. rhamnosus* 1; one was not statistically significant. Generally, the ability to stimulate the growth was higher in relation to *L. rhamnosus* 1 compared to that in the case of *L. acidophilus*. Based on the findings, the mushrooms can be divided into two groups, one group with activities from 10 to 30% and the other with activities above 30%. *P. capnoides* was found to possess the highest activity (56.63%) while *P. betulinus* performed the worst among the mushrooms that exhibited activity (10.98%). Surprisingly, *P. mutabilis*, which was not active in the case of *L. acidophilus*, gave a high activity of 49.22%. Polysaccharides from the edible species *M. procera* and *S. crispa* demonstrated high stimulation of lactobacilli growth, confirming their potential using as nutraceuticals. FOS and inulin showed similar activities, 11.71% and 12.72%, respectively, values lower than those obtained for majority of investigated polysaccharides in relation to *L. rhamnosus* 1.

The clinical strain of *L. rhamnosus* 2 was less susceptible to growth using mushroom polysaccharides. Twenty-one of the thirty-five investigated polysaccharides were found to be statistically significant in enhancing the growth of *L. rhamnosus* 2. The highest result was obtained for *C. cinnamomeus* (17.84%) and the lowest for *B. adusta* (3.93%). Generally, most of the analyzed species revealed activities ranging between 10% and 15%. The activities of FOS and inulin were 11.34 and 8.87%, respectively, which were again lower than the activities of the mushroom polysaccharides.

### Resistance toward acid digestibility

**Fig. 4:**
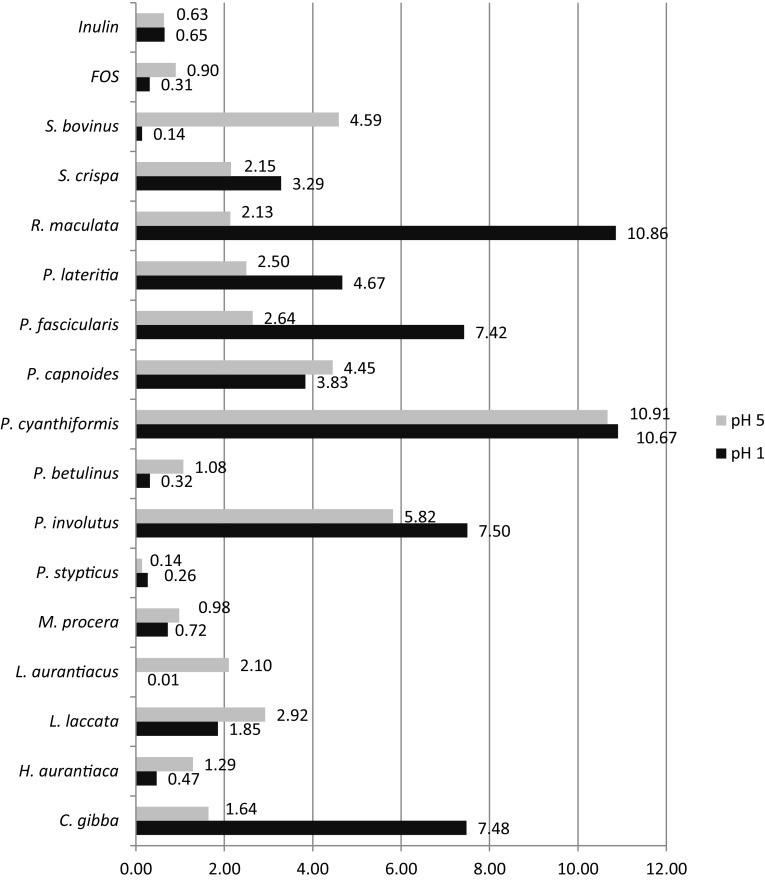
Degree of polysaccharide hydrolysis upon exposure to artificial human gastric juice at pH values of 1 and 5 [%]

The degree of hydrolysis for the polysaccharides, inulin and FOS in artificial gastric juice are shown in Fig. 4. The values obtained in pH 1 range from 0.01% for *L. aurantiacus* to 10.86% for *R. maculata*, while in pH 5, the values range from 0.14 to 10.91% for *P. stypticus* and *P. cyanthiformis*, respectively. Inulin was found to be hydrolyzed similarly in both pH values, but FOS revealed greater resistance to digestion in a pH of 1 than a pH of 5. Generally, the mushroom polysaccharides investigated remained more than 90% undigested, except in the cases of *P. cyanthiformis* and *R. maculata*. These results suggest that polysaccharides are able to pass through the stomach in unchanged form to reach the colon and stimulate the growth of beneficial bacteria. The differences in hydrolysis under different pH conditions may result from the structural composition of polysaccharides extracted from mushrooms and their possible connections to other compounds.

## Discussion

Several studies have indicated that probiotic bacteria are capable of utilizing galacto-, manno-, and fructooligosaccharides, as well as other carbohydrate compounds, including inulin, lactulose and lactitol [23]. Due to their chemical structure, non-digestible carbohydrates are neither absorbed in the upper gastrointestinal tract nor hydrolyzed by human enzymes. Therefore, they are excellent substrates for colonic bacteria [24]. Currently, prebiotic sugars are produced in large scale from different natural sources. However, most of the products available on the market are based on inulin. In addition to their health benefits, such as enhancement of gastrointestinal health and the immune system, reduction of cancer risk, anti-inflammatory effects, decline of osteoporosis and anti-obesity effects, prebiotics are useful in food processing because they improve the organoleptic properties of food products and provide enhanced nutritional value [11]. Prebiotics have gelling properties and can replace fat in some products without loss of texture. They can also increase the freshness of bakery products. Generally, utilization of prebiotic ingredients in functional food, including dairy products and breads, is preferable because they increase the viability of probiotic bacteria [25]. The above facts suggest that polysaccharides from edible mushrooms could also be used as food ingredients. Therefore, we examined whether mushrooms containing large amounts of polysaccharides could be a potential source of growth substrates for probiotic bacteria strains.

The first aim of our research was to obtain the maximum possible quantities of polysaccharides from the wild-growing mushrooms. The extraction of polysaccharides from mushrooms is usually conducted with hot water. However, the yield of this process depends on different variables, such as temperature, time, the water/solid ratio and the number of extractions. According to Zhang et al., the effects of the factors on the extraction yield of polysaccharides from *Flammulina velutipes* were ordered as follows: extraction temperature > ratio of water to raw material > extraction time [26]. The yield of polysaccharides obtained for this species by conventional hot water extraction varied from 6.98 to 9.68%. Using ultrasound-assisted extraction at a lower temperature and a shorter time gave a slight boost to yield (8.76–11.46%). According to the study on effects of the extraction method on polysaccharides of *Clitocybe maxima*, the mean yield using a hot water extraction was 5.86% [27]. These results suggest that novel methods of extraction, including ultrasonic-assisted extraction, can be more efficient. Furthermore, previous studies have demonstrated that ultrasonic-assisted extraction, which was also applied in this study, possesses several advantages: it accelerates the extraction process, causes less damage to structural and molecular properties and requires lower temperature [28].

The screening study was conducted to determine whether the probiotic strains of *L. acidophilus* and *L. rhamnosus* were able to utilize polysaccharides extracted from the fruiting bodies of mushrooms. Several previous studies have demonstrated that polysaccharides extracted from *Pleurotus* spp., L*entinus edodes, Tremella fuciformis* and *Agaricus bisporus* can be used as nutraceuticals [29–31]. According to Synytsya et al., polysaccharides isolated from *P. ostreatus* and *P. eryngii* enhance the growth rate of probiotic *Lactobacillus* bacteria [29]. Yu et al. have reported that polysaccharopeptide (PSP) from *Trametes versicolor* promotes the growth and activity of probiotic bacterial genera, such as *Bifidobacteria* and *Lactobacilli* [32]. An *in vivo* study regarding the effect of PSP from *T. versicolor* on the gut microbiome of healthy volunteers confirmed that this compound acts as a prebiotic and modulates the human intestinal microbiome composition [33]. According to another study, polysaccharides extracted from such mushroom wastes as *Lentinula edodes* stipe, *Pleurotus eryngii* base, and *Flammulina velutipes* with boiling water, trichloroacetic acid precipitation and ethanol precipitation enhanced the survival rate of *Lactobacillus acidophilus, Lactobacillus casei* and *Bifidobacterium longum* subsp. *longum* during cold storage [34]. Gao et al. demonstrated that β-glucan-type polysaccharides from two medicinal mushrooms, *Poria cocos* and *Polyporus rhinoceros*, stimulate the growth of some beneficial bacteria [35]. A precise explanation of the structure–function relationship, including the determination of molecular weights and contribution of glycosidic linkages, would provide crucial insight into the utilization of these polysaccharides by probiotic bacteria. Several in vitro research studies and animal experiments from the late 1990s and early 2000s have indicated that β-glucans from oat and barley selectively promote the growth of lactobacilli and bifidobacteria; active studies on β-glucans as novel prebiotics are still being conducted [36]. Therefore, our research was an attempt to find new sources of such compounds in mushrooms. The fungal polysaccharides revealed stronger activity than inulin and FOS, so further extensive studies are necessary to develop food supplements or nutraceuticals that constitute growth substrate for probiotics from mushrooms.

The concept of prebiotics is laid out by certain criteria, including resistance to gastric acidity, to ensure they reach the colon in order to effectively stimulate probiotic bacteria [37]. Therefore, selected mushroom polysaccharides were subjected to artificial human gastric juice to estimate their level of digestibility in the stomach. Food is usually retained in the human stomach for approximately 2 h under acidic conditions; in further sections of the GI tract, the pH value rises to approximately 5 [17, 38].

There is a lack of knowledge regarding the digestibility of mushroom polysaccharides in humans. According to the available data, even though mushroom polysaccharides possess different chemical composition, they are mainly β-glucans. β-Glucosidic bonds are resistant to acid hydrolysis in the stomach, so fungi polysaccharides could be considered potential prebiotics [3]. However, more detailed studies are required on the non-digestive properties of mushroom carbohydrates.

## Conclusions

Our findings indicate that mushrooms contain significant amounts of polysaccharides that were found to enhance the growth of lactobacilli. The majority of the polysaccharides studied stimulated the growth of the reference strain *L. acidophilus* and two strains of *L. rhamnosus* isolated from the human gastrointestinal tract. Several edible mushroom species could be recommended for enhancing the number of beneficial bacteria in the GI tract. The polysaccharide fractions from mushrooms were resistant to gastric acidity, which ensures that they can reach the colon to effectively stimulate probiotic bacteria. More detailed studies are needed to determine the way that probiotic strains utilize mushroom polysaccharides as well as the metabolic pathways activated in this process. The fragmentation of mushroom polysaccharides into smaller molecules is likely to make them easily accessible for utilization as a source of energy by probiotics. However, our screening study of 53 mushroom polysaccharides revealing that these compounds constitute effective growth substrate for *Lactobacillus* strains encourages future research into structural characterization of the polysaccharide extracts and *in vivo* experiments. There is a huge diversity of mushroom species under investigation as potential sources of biologically active compounds. The majority of species presented in our paper are investigated for the first time; the findings provided some basis for the comprehensive study of mushrooms growing wild in Poland.

## Electronic supplementary material

Below is the link to the electronic supplementary material.

Supplementary material 1 (DOCX 114 KB)
