# Supplementary material for: The preliminary study of prebiotic potential of Polish wild mushroom polysaccharides: the stimulation effect on Lactobacillus strains growth
Source: Eur J Nutr. 2017 Mar 28;57(4):1511–21. doi: 10.1007/s00394-017-1436-9 (PMC5959981; doi:10.1007/s00394-017-1436-9)
Supplement: Supplementary file 1 — Supplementary material 1 (DOCX 114 KB) [file 394_2017_1436_MOESM1_ESM.docx]

**The preliminary study of prebiotic potential of polish wild mushroom polysaccharides - the stimulation effect on *Lactobacillus* strains growth.**

**European Journal of Nutrition**

**Renata Nowak ^a*^, Natalia Nowacka-Jechalke ^a^, Marek Juda ^b^, Anna Malm ^b^**

^a^ Chair and Department of Pharmaceutical Botany, Medical University of Lublin, 1 Chodźki Street, 20-093 Lublin, Poland

^b^ Department of Pharmaceutical Microbiology, Medical University of Lublin, 1 Chodźki Street, 20-093 Lublin, Poland

* Corresponding author. Tel.: +48 81 742 37 03; fax: +48 81 742 38 05

E-mail address: renata.nowak@umlub.pl


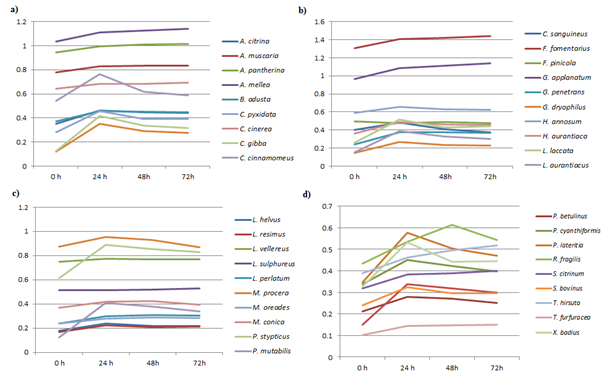


**Fig. 2** Absorbance values at incubation hour 0, 24, 48 obtained in the determination of growth stimulation activity of mushroom polysaccharides.

**Tab. 1** Absorbance values obtained in determination of growth stimulation activity of mushroom polysaccharides and controls after 48 h incubation. Species demonstrated statistical significance (p ≤ 0.05) were subjected for further analysis.

| **Species** | ***L. acidophilus*** | | | ***L. rhamnosus* 1** | | | ***L. rhamnosus* 2** | | |
| --- | --- | --- | --- | --- | --- | --- | --- | --- | --- |
|  | **Absorbance**  **±SD** | **Control**  **absorbance**  **±SD** | ***p-*values** | **Absorbance ±SD** | **Control absorbance ±SD** | ***p-*values** | **Absorbance ±SD** | **Control absorbance ±SD** | ***p-*values** |
| *A. citrina* | 0.445±0.014 | 0.355±0.010 | 0.000827 | 0.393±0.007 | 0.170±0.016 | 0.000028 | 0.294±0.038 | 0.174±0.025 | 0.010452 |
| *A. muscaria* | 0.835±0.023 | 0.751±0.010 | 0.004349 | 0.580±0.002 | 0.308±0.047 | 0.000553 | 0.397±0.008 | 0.317±0.034 | 0.015818 |
| *A. pantherina* | 1.009±0.009 | 0.945±0.028 | 0.1137 | - | - | - | - | - | - |
| *A. mellea* | 1.128±0.020 | 1.021±0.010 | 0.001133 | 0.597±0.020 | 0.409±0.018 | 0.000289 | 0.573±0.011 | 0.413±0.008 | 0.017584 |
| *B. adusta* | 0.450±0.015 | 0.38±0.014 | 0.004136 | 0.624±0.010 | 0.377±0.024 | 0.028241 | 0.422±0.002 | 0.379±0.018 | 0.014679 |
| *C. pyxidata* | 0.390±0.084 | 0.293±0.009 | 0.1168 | - | - | - | - | - | - |
| *C. cinerea* | 0.685±0.009 | 0.649±0.010 | 0.008 | 0.447±0.012 | 0.483±0.024 | - | 0.464±0.035 | 0.486±0.125 | - |
| *C. gibba* | 0.338±0.012 | 0.151±0.007 | 0.000075 | - | - | - | - | - | - |
| *C. nebularis* | 0.327±0.005 | 0.345±0.011 | - | - | - | - | - | - | - |
| *C. micaceus* | 0.566±0.012 | 0.588±0.003 | - | - | - | - | - | - | - |
| *C. cinnamomeus* | 0.617±0.028 | 0.536±0.013 | 0.00924 | 0.674±0.026 | 0.225±0.026 | 0.000029 | 0.422±0.112 | 0.227±0.029 | 0.042537 |
| *C. sanguineus* | 0.412±0.024 | 0.403±0.013 | 0.4644 | 0.480±0.008 | 0.214±0.02 | 0.000026 | 0.334±0.017 | 0.242±0.003 | 0.019002 |
| *D. confragosa* | 1.178±0.036 | 1.283±0.010 | - | - | - | - | - | - | - |
| *F. fomentarius* | 1.419±0.015 | 1.336±0.008 | 0.000963 | 0.806±0.019 | 0.533±0.144 | 0.031368 | 0.618±0.013 | 0.632±0.069 | - |
| *F. pinicola* | 0.488±0.004 | 0.447±0.008 | 0.001057 | - | - | - | - | - | - |
| *G. applanatum* | 1.114±0.019 | 1.06±0.009 | 0.0124 | - | - | - | - | - | - |
| *G. penetrans* | 0.375±0.011 | 0.255±0.006 | 0.000088 | 0.449±0.036 | 0.161±0.016 | 0.000231 | 0.320±0.078 | 0.167±0.033 | 0.035361 |
| *G. dryophilus* | 0.235±0.010 | 0.158±0.007 | 0.000508 | 0.476±0.01 | 0.080±0.025 | 0.000014 | 0.234±0.018 | 0.064±0.023 | 0.000509 |
| *H. annosum* | 0.630±0.024 | 0.587±0.019 | 0.04359 | - | - | - | - | - | - |
| *H. aurantiaca* | 0.465±0.004 | 0.368±0.010 | 0.000099 | 0.379±0.018 | 0.226±0.029 | 0.00153 | 0.237±0.002 | 0.237±0.058 | - |
| *H. paradoxa* | 1.161±0.037 | 1.441±0.007 | - | - | - | - | - | - | - |
| *L. amethystea* | 0.797±0.024 | 0.819±0.005 | - | - | - | - | - | - | - |
| *L. laccata* | 0.429±0.008 | 0.281±0.017 | 0.000147 | 0.706±0.081 | 0.157±0.086 | 0.0013 | 0.287±0.005 | 0.195±0.058 | 0.018099 |
| *L. aurantiacus* | 0.331±0.010 | 0.191±0.007 | 0.000036 | 0.509±0.087 | 0.109±0.015 | 0.001406 | 0.275±0.031 | 0.174±0.075 | 0.097603 |
| *L. helvus* | 0.221±0.004 | 0.173±0.007 | 0.000665 | 0.523±0.02 | 0.088±0.013 | 0.000006 | 0.225±0.006 | 0.117±0.024 | 0.001523 |
| *L. resimus* | 0.212±0.004 | 0.185±0.018 | 0.012015 | - | - | - | - | - | - |
| *L. rufus* | 1.327±0.097 | 1.357±0.010 | - | - | - | - | - | - | - |
| *L. vellereus* | 0.773±0.007 | 0.751±0.012 | 0.0673 | 0.658±0.004 | 0.310±0.022 | 0.000011 | 0.409±0.013 | 0.330±0.024 | 0.007335 |
| *L. sulphureus* | 0.521±0.013 | 0.481±0.008 | 0.000293 | 0.450±0.05 | 0.308±0.018 | 0.01033 | 0.367±0.053 | 0.310±0.053 | 0.258403 |
| *L. scabrum* | 1.187±0.009 | 0.196±0.008 | - | - | - | - | - | - | - |
| *L. flaccida* | 0.309±0.014 | 0.331±0.011 | - | - | - | - | - | - | - |
| *L. perlatum* | 0.309±0.007 | 0.228±0.007 | 0.000182 | 0.481±0.012 | 0.125±0.035 | 0.000077 | 0.280±0.022 | 0.107±0.052 | 0.006168 |
| *M. procera* | 0.932±0.005 | 0.834±0.013 | 0.000279 | 0.639±0.052 | 0.334±0.032 | 0.000988 | 0.358±0.038 | 0.365±0.079 | - |
| *M. oreades* | 0.289±0.011 | 0.25±0.008 | 0.009726 | 0.416±0.013 | 0.127±0.028 | 0.000082 | 0.266±0.001 | 0.142±0.053 | 0.015434 |
| *M. conica* | 0.424±0.011 | 0.357±0.010 | 0.001393 | 0.506±0.007 | 0.156±0.031 | 0.000045 | 0.338±0.008 | 0.159±0.029 | 0.000347 |
| *P. stypticus* | 0.856±0.025 | 0.691±0.008 | 0.000424 | 0.456±0.011 | 0.251±0.015 | 0.000048 | 0.399±0.037 | 0.251±0.049 | 0.013708 |
| *P. involutus* | 2.591±0.064 | 2.394±0.006 | 0.006242 | 0.784±0.023 | 0.894±0.131 | - | 0.885±0.01 | 0.895±0.128 | - |
| *P. mutabilis* | 0.380±0.022 | 0.364±0.009 | 0.1361 | 0.613±0.009 | 0.075±0.021 | 0.000002 | 0.242±0.008 | 0.677±0.087 | - |
| *P. betulinus* | 0.272±0.011 | 0.171±0.005 | 0.000052 | 0.198±0.021 | 0.078±0.025 | 0.003308 | 0.173±0.007 | 0.173±0.007 | - |
| *P. cyanthiformis* | 0.424±0.033 | 0.296±0.010 | 0.002767 | 0.454±0.071 | 0.130±0.026 | 0.001787 | 0.312±0.035 | 0.148±0.051 | 0.010088 |
| *P. capnoides* | 0.266±0.008 | 0.108±0.004 | 0.000007 | 0.694±0.169 | 0.075±0.017 | 0.003186 | 0.244±0.001 | 0.128±0.064 | 0.036317 |
| *P. fascicularis* | 0.314±0.008 | 0.143±0.004 | 0.000005 | 0.601±0.001 | 0.092±0.008 | 0.000056 | 0.221±0.017 | 0.095±0.028 | 0.002746 |
| *P. lateritia* | 0.505±0.015 | 0.303±0.012 | 0.000053 | 0.542±0.059 | 0.349±0.219 | 0.214249 | 0.255±0.057 | 0.143±0.052 | 0.066437 |
| *R. maculata* | 0.368±0.092 | 0.094±0.007 | 0.003231 | 0.605±0.008 | 0.081±0.023 | 0.000003 | 0.226±0.011 | 0.086±0.017 | 0.000286 |
| *R. fragilis* | 0.614±0.117 | 0.551±0.007 | 0.406 | - | - | - | - | - | - |
| *S. citrinum* | 0.390±0.014 | 0.313±0.022 | 0.01 | 0.457±0.03 | 0.248±0.046 | 0.002678 | 0.422±0.012 | 0.253±0.053 | 0.005504 |
| *S. crispa* | 0.221±0.013 | 0.113±0.007 | 0.000224 | 0.559±0.024 | 0.063±0.022 | 0.000013 | 0.183±0.007 | 0.068±0.03 | 0.003176 |
| *S. bovinus* | 0.296±0.033 | 0.178±0.006 | 0.000296 | 0.277±0.033 | 0.154±0.046 | 0.019491 | 0.196±0.034 | 0.129±0.028 | 0.058323 |
| *S. variegatus* | 0.310±0.019 | 0.328±0.009 | - | - | - | - |  |  |  |
| *T. hirsuta* | 0.495±0.029 | 0.416±0.008 | 0.01 | - | - | - |  |  |  |
| *T. versicolor* | 0.275±0.003 | 0.275±0.008 | - | - | - | - |  |  |  |
| *T. furfuracea* | 0.148±0.008 | 0.09±0.004 | 0.000337 | - | - | - |  |  |  |
| *X. badius* | 0.443±0.011 | 0.459±0.009 | - | 0.411±0.06 | 0.152±0.036 | 0.002968 | 0.182±0.003 | 0.162±0.032 | 0.340902 |
| Inulin | 0.200±0.002 | 0.076±0.009 | 0.000016 | 0.226±0.015 | 0.087±0.014 | 0.000317 | 0.185±0.002 | 0.088±0.016 | 0.000458 |
| FOS | 0.174±0.004 | 0.048±0.006 | 0.000006 | 0.192±0.007 | 0.064±0.03 | 0.001987 | 0.165±0.004 | 0.041±0.016 | 0.00017 |
